# Electrochemical Synthesis of Aminated Polyaniline/Multi-Walled Carbon Nanotube Composite for Selective Dopamine Detection in Artificial Urine

**DOI:** 10.3390/polym17182539

**Published:** 2025-09-19

**Authors:** Saengrawee Sriwichai, Pimmada Thongnoppakhun

**Affiliations:** 1 Department of Chemistry, Faculty of Science, Chiang Mai University, Chiang Mai 50200, Thailand; thongnoppakhunpimmada@gmail.com; 2 Center of Excellence in Materials Science and Technology, Chiang Mai University, Chiang Mai 50200, Thailand; 3 Center of Excellence for Innovation in Chemistry (PERCH-CIC), Faculty of Science, Chiang Mai University, Chiang Mai 50200, Thailand

**Keywords:** conducting polymer, carbon nanotube, dopamine, sensor, composite

## Abstract

Monitoring dopamine (DA) has attracted increasing attention due to alterations in DA levels associated with brain disorders. In addition, the urinary DA concentration plays a significant role in the sympathoadrenal system. A decrease in DA can impair reward-seeking behavior and cognitive flexibility. Therefore, accurate and precise DA detection is necessary. In this study, a poly(3-aminobenzylamine)/functionalized multi-walled carbon nanotube (PABA/f-CNT) composite thin film was fabricated by electrochemical synthesis, or electropolymerization, of 3-aminobenzylamine (3-ABA) monomer and f-CNTs through cyclic voltammetry (CV) on a fluorine-doped tin oxide (FTO)-coated glass substrate, which also served as a working electrode for label-free DA detection in artificial urine. The formation of the film was confirmed by the obtained cyclic voltammogram, electrochemical impedance spectroscopy (EIS) plots, and scanning electron microscope (SEM) and transmission electron microscope (TEM) images. The chemical components of the films were analyzed using attenuated total reflection–Fourier transform infrared (ATR–FTIR) spectroscopy and X-ray photoelectron spectroscopy (XPS). For label-free DA detection, various concentrations (50–1000 nM) of DA were determined in buffer solution through differential pulse voltammetry (DPV). The fabricated PABA/f-CNT film presented two linear ranges of 50–400 nM (R^2^ = 0.9915) and 500–1000 nM (R^2^ = 0.9443), with sensitivities of 1.97 and 0.95 µA·cm^−2^·µM^−1^, respectively. The limit of detection (LOD) and the limit of quantity (LOQ) were 119.54 nM and 398.48 nM, respectively. In addition, the PABA/f-CNT film provided excellent selectivity against common interferents (ascorbic acid, uric acid, and glucose) with high stability, reproducibility, and repeatability. For potential future medical applications, DA detection was further performed in artificial urine, yielding a high percentage of recovery.

## 1. Introduction

The monitoring of dopamine with a non-invasive approach, i.e., detection in body fluids such as saliva, urine, sweat, and tears, has been shown to be suitable for the measurement of analytes without the need for active monitoring, e.g., blood extraction, which is often inconvenient for many patients [1,2]. The key catecholamine neurotransmitter, dopamine (DA) or 3,4-dihydroxyphenyl ethylamine, plays a significant role in the sympathoadrenal system when present in urine [3,4]. In biological systems or organisms, normal DA concentrations range from 0.1 μM to 1 mM [5]. In a healthy person, the urinary DA concentration is very low, ranging from 274 to 500 nmol/L [6]. Fluctuations in DA levels are implicated in various neurological diseases, for example, depression, fatigue in multiple sclerosis, psychiatric disorders, and other neurological disorders [7,8]. Hence, the monitoring of DA with accuracy and precision is necessary. Several common analytical methods, including fluorescence spectroscopy, capillary electrophoresis, high-performance liquid chromatography, and gas chromatography, have recently been studied to measure DA in human biological systems [9,10,11]. However, these conventional methods require large and high-cost instruments, as well as time-consuming pretreatment of biological samples. Electrochemical measurements provide an attractive alternative, offering simplicity, rapid analysis, and real-time monitoring [12,13,14,15,16,17]. Consequently, the development of a straightforward and simple-to-use sensor for DA detection based on an electrochemical method has been intensively and extensively considered. Electrochemical methods offer simplicity, cost-effectiveness, high performance, and real-time monitoring [15,18]. Electrochemical sensors, including conventional electrochemical sensors, such as potentiometric sensors [19,20] and electrochemical impedance spectroscopy (EIS)-based sensors [21,22], have been widely investigated. Furthermore, working electrode modification with conductive materials is one of the most effective pathways for developing DA sensors with good sensitivity and selectivity.

Conducting polymers, such as polypyrroles, polyanilines, and polythiophenes, are highly conductive and electroactive materials with excellent electrical and optical properties, effective redox properties, high conductivity, and stability with biocompatibility [23]. Among them, polyaniline and its derivatives have been extensively studied for various applications, including electrochemical sensors. Conducting polymers can be synthesized using several methods, including electrochemical polymerization or electropolymerization, chemical polymerization, photochemical polymerization, and solid-state polymerization [24,25,26,27]. Among these synthetic methods, electrochemical polymerization, or electropolymerization, is a particularly convenient method due to its simplicity, low cost, and efficiency, allowing direct polymerization onto the electrode surface [28,29,30,31]. Hence, conducting polymer-based electrochemical sensors, in which the analytes interact with the polymers to produce an electrochemical signal, have gained much attention due to their high sensitivity and facile operation at room temperature [23,32]. In addition, composites of conducting polymers with other conductive materials, e.g., carbon nanotubes (CNTs) and graphene oxide [33,34], gold nanoparticles [35], and MoO_3_ [36], have received considerable attention for application in electrochemical sensors, especially DA sensors. Carbon-based nanomaterials, e.g., fullerenes, graphene, graphene oxide, graphene nanoplates, and CNTs, exhibit remarkable mechanical, thermal, and electrical properties [33,37,38,39,40]. Graphenes consist of a two-dimensional crystalline sheet structure with in-plane hexagonal rings of carbon atoms. In contrast, carbon nanotubes (CNTs) exhibit a cylindrical structure, resembling a rolled-up graphene sheet with a nanoscale diameter. Fullerene (C-60) is a zero-dimensional, cage-like spherical molecule composed of 20 hexagons and 12 pentagons [40]. Among these, CNTs, discovered by Ijima in 1991 [41], have been the most representative nanomaterials [40,42] due to their ability to improve the electrical conductivity and mechanical properties of CNT-based nanomaterials. CNTs are generally categorized into single-walled carbon nanotubes and multi-walled carbon nanotubes, typically synthesized by carbon-arc discharge, laser ablation of carbon, or chemical vapor deposition [43]. The main advantages of multi-walled carbon nanotubes over single-walled carbon nanotubes include lower cost, reduced defect sensitivity, and easier synthesis with high purity [43,44], making them more practical for a wide range of applications. Therefore, multi-walled carbon nanotubes were chosen in the present study. In addition, functionalized carbon nanotubes (f-CNTs) have been widely employed for the promotion of electron transfer and increasing the electrode surface area with improved dispersion and compatibility with conducting polymers [13,15]. Hence, f-CNTs were incorporated to improve the properties of aminated polyaniline derivative in this study.

**Scheme 1:**
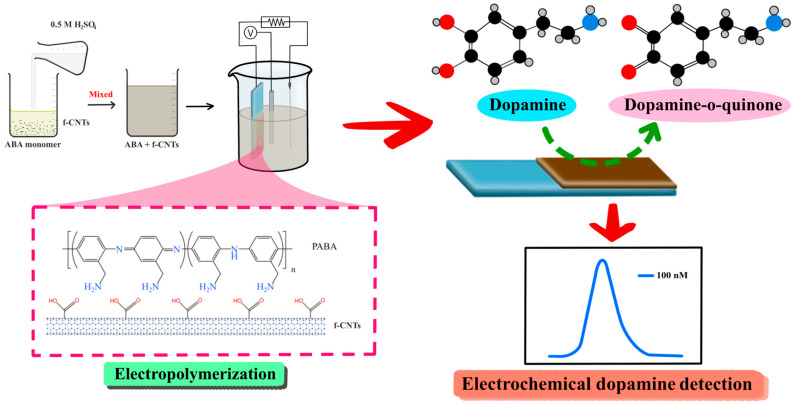
Schematic diagram of the electrochemical synthesis of PABA/f-CNT (**left panel**) for DA detection (**right panel**).

We recently reported the use of electrospun PABA/f-CNT nanofibers for the electrochemical detection of DA [15]. However, the electrospinning technique is time-intensive. Therefore, the present research work aimed to fabricate a composite film of poly(3-aminobenzylamine) (PABA) and f-CNTs via electrochemical synthesis for selective DA detection in artificial urine. The novelty of this work lies in the electrochemical preparation of a PABA/f-CNT composite, offering a simple, time-efficient fabrication process and enabling the detection of trace amounts of DA. The PABA/f-CNT composite film was synthesized electrochemically through cyclic voltammetry (CV) in a mixture of 3-aminobenzylamine monomer and f-CNTs in 0.5 M H_2_SO_4_ on a fluorine-doped tin oxide (FTO)-coated glass substrate. The characterization of the obtained composite film was performed by scanning electron microscopy (SEM), transmission electron microscopy (TEM), X-ray photoelectron spectroscopy (XPS), and attenuated total reflectance–Fourier transform infrared (ATR-FTIR) spectroscopy. The electrochemical activity of the composite film was examined using EIS and CV prior to use as the working electrode for DA detection. To study the detection performance, the electrochemically synthesized PABA/f-CNT film was employed as the working electrode for electrochemical DA detection with differential pulse voltammetry (DPV). The process of electrochemical synthesis of the PABA/f-CNT composite on the FTO-coated glass substrate as the working electrode for selective DA detection is presented in Scheme 1.

## 2. Materials and Methods

### 2.1. Materials

All chemicals were used as received without further purification. Dopamine (DA), 3-aminobenzyl amine (ABA), glucose (Glu), potassium chloride (KCl), phosphate-buffer saline (PBS) tablets, and FTO-coated glass substrate (resistivity ~8 Ω/sq) were purchased from Sigma-Aldrich (Darmstadt, Germany). Ascorbic acid (AA) was purchased from Poch (Gliwice, Poland). Uric acid (UA) was acquired from Bio Basic (Markham, ON, Canada). Potassium hexacyanoferrate (K_3_Fe(CN)_6_) was acquired from Scharlau (Barcelona, Spain). Artificial urine was acquired from Pickering Laboratories (Mountain View, CA, USA). Aqueous PBS solution containing K_3_Fe(CN)_6_ was prepared in DI water. The FTO-coated glass substrate was cleaned with DI water and ethanol in an ultrasonic bath. The f-CNTs were prepared using a mixture of nitric acid and sulfuric acid (1:3 *v*/*v*), as previously reported [13].

### 2.2. Instrumentation

ATR-FTIR spectroscopy (Bruker Tensor 27, Billerica, MA, USA) and X-ray photoelectron spectroscopy (XPS; AXIS ultra DLD spectrometer, Manchester, UK) were employed to investigate the chemical constituents of the fabricated films. The surface morphology was examined using transmission electron microscopy (TEM; JEOL JEM-2010, Tokyo, Japan) and scanning electron microscopy (SEM; JEOL JSM–6335F, Tokyo, Japan). The CV, DPV, and EIS data were obtained by performing electrochemical experiments using PalmSens4 potentiostat/galvanostat with an optional impedance analyzer in the 3-electrode system. The FTO-coated glass substrate was used as the working electrode, Ag/AgCl (in 3 M KCl) was used as the reference electrode, and a platinum wire was used as the counter electrode in this work. All reported potentials in this study were relative to this Ag/AgCl reference electrode.

### 2.3. Electrochemical Synthesis of PABA/f-CNT Composite

Electrochemical synthesis, illustrated in the left panel of Scheme 1, was performed using the ABA composite mixture (15 mM) and f-CNT (0.001% *w*/*v*) in 0.5 M H_2_SO_4_ at a scan rate of 20 mV/s under a potential range of 0–1.1 V for 10 cycles. The resulting PABA/f-CNT electrode was washed with 0.5 M H_2_SO_4_, followed by DI water. The chemical constituents of the electrode were investigated using XPS and ATR-FTIR spectroscopy. TEM and SEM were performed for studying the morphology of the electrode. Prior to use for selective and label-free electrochemical DA detection, the electrochemical characteristics were studied using the CV technique in the potential range of −0.2–0.9 V for 1 cycle in an aqueous solution of PBS containing 0.5 mM K_3_Fe(CN)_6_ and 0.1 M KCl at various scan rates (10–100 mV/s). EIS measurements were also performed in the PBS containing 0.5 mM K_3_Fe(CN)_6_ and 0.1 M KCl to assess the surface resistance of the electrode at a frequency range of 1 Hz to 1 MHz at open circuit potential.

### 2.4. Electrochemical Selective Dopamine Detection

As shown in the right panel of Scheme 1, the prepared PABA/f-CNT electrode was employed for selective dopamine detection in the PBS containing 0.5 mM K_3_Fe(CN)_6_ and 0.1 M KCl using a DPV technique in the potential range of −0.1–0.9 V at a scan rate of 5 mV/s. For the sensitivity investigation, successive DA concentrations of 50–1000 nM were added to the PBS solution. The selectivity experiment was performed by adding common interferents, i.e., UA, AA, and glucose (1 mM) to the PBS solution. The reproducibility of the electrode was investigated using three independently electrochemically synthesized electrodes by adding 0.1 mM DA to the PBS solution. To examine the repeatability of the electrode, DPV scans were performed for up to 10 cycles with successive additions of 0.1 mM DA to the PBS buffer solution. The stability of the electrode was demonstrated at 2-day intervals over 7 days by performing DPV using 0.1 mM DA in the PBS solution. The electrodes were stored under a dry atmosphere prior to testing. In addition, to verify the reliability of this electrode for practical applications in the future, the PABA/f-CNT electrode was used for the selective detection of DA in 5 % *v*/*v* artificial urine in the PBS solution. The DPV responses were recorded using three different electrodes for each DA concentration (350, 550, and 750 nM for PABA and 50, 150, 250, 350, 550, and 750 nM for PABA/f-CNTs). The percentage of recovery in the artificial urine was calculated from the DPV peak responses of each DA concentration [15].

## 3. Results and Discussion

### 3.1. Characterization of PABA/f-CNT Electrode

**Figure 1:**
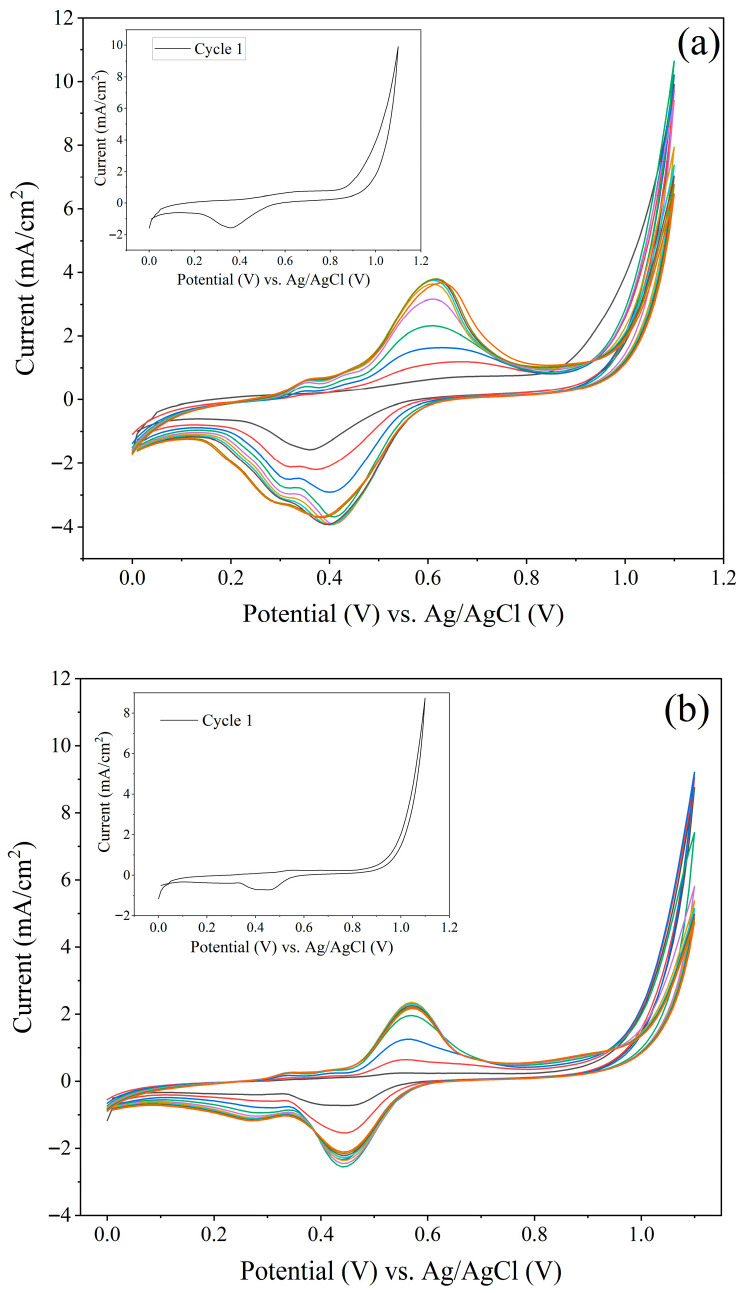
Cyclic voltammograms for electrochemical synthesis of (**a**) PABA and (**b**) PABA/f-CNTs on FTO electrodes in 0.5 M H_2_SO_4_ at a scan rate of 20 mV/s for 10 cycles.

Cyclic voltammograms for the electrochemical synthesis of PABA/f-CNT and PABA films on FTO electrodes in 0.5 M H_2_SO_4_ at a scan rate of 20 mV/s under a potential range of 0–1.1 V for 10 cycles are displayed in Figure 1. As observed in the inset of Figure 1a,b, the first cycle of the cyclic voltammogram presented a cathodic peak at about 0.35 V for the PABA film and about 0.45 V for the PABA/f-CNTs. After the second cycle of electropolymerization, both PABA/f-CNTs and PABA showed two oxidation peaks at about 0.35 and 0.6 V with an additional cathodic peak at about 0.3 V. These results indicate that both PABA and PABA/f-CNTs, where PABA is an aminated derivative of polyaniline, presented the characteristic electrochemical behavior of polyaniline [31].

**Figure 2:**
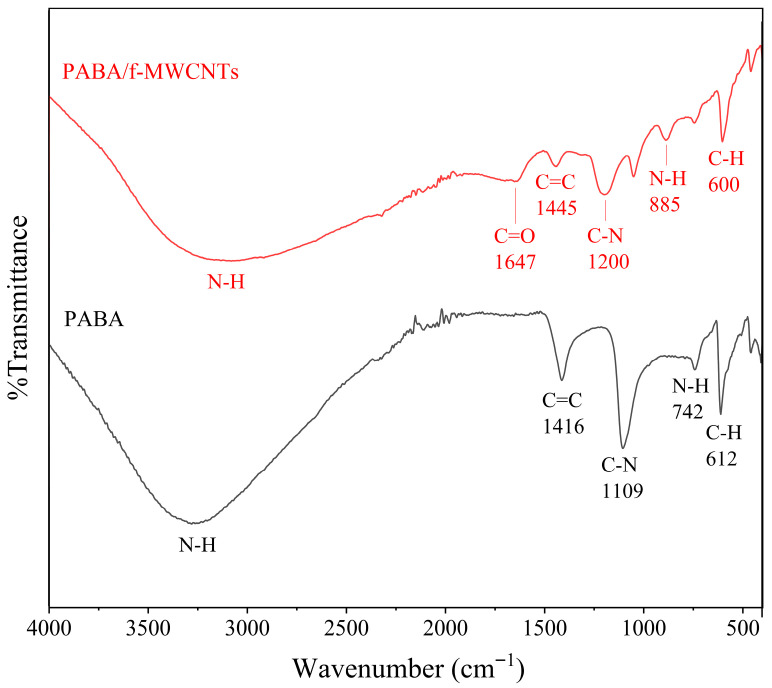
ATR-FTIR spectra of electropolymerized PABA/f-CNT and PABA films.

The electrochemically synthesized PABA/f-CNTs and PABA on FTO electrodes were characterized by XPS, ATR-FTIR spectroscopy, TEM, and SEM. The chemical constituents of the electrodes were analyzed by ATR-FTIR spectroscopy and XPS. Figure 2 presents the ATR-FTIR spectra of the electrochemically synthesized PABA/f-CNTs and PABA on FTO electrodes. The essential C–O stretching peak of the carboxylic group (COOH) on the surface of f-CNTs was clearly seen at about 1650 cm^−1^ in the PABA/f-CNT film [15]. C-C stretching of the aromatic ring in the PABA structure presented peaks at 1416 and 1445 cm^−1^ for the PABA and PABA/f-CNT composite, respectively [14]. The broad peaks at 3100–3300 cm^−1^ are attributed to N-H stretching of the amine group in the PABA. In addition, C-N stretching peaks at 1200 and 1109 cm^−1^ for the PABA/f-CNTs and PABA, respectively, were also observed. N-H wagging peaks appeared at 885 and 742 cm^−1^ for the PABA/f-CNTs and PABA, respectively. Shifts in these peaks indicate an interaction between the PABA and f-CNTs in the composite film. Identical spectra were observed for both electrodes, except that the carboxylic acid group of f-CNTs only appeared in the PABA/f-CNT electrode. These results confirm the successful formation of PABA/f-CNTs on the FTO electrode.

**Figure 3:**
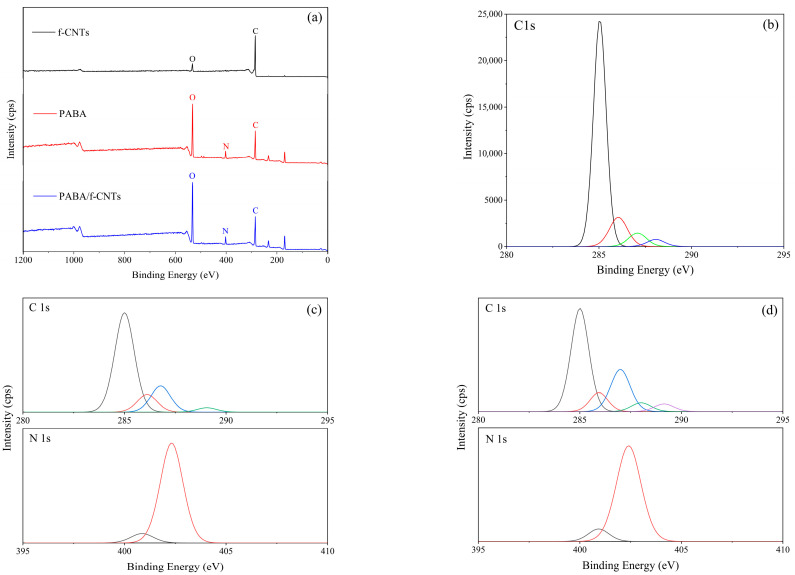
XPS (**a**) survey spectra with high-resolution spectra of (**b**) f-CNTs, (**c**) PABA, and (**d**) PABA/f-CNT films.

For further analysis of the chemical composition of the obtained electropolymerized PABA and PABA/f-CNT films, the XPS spectra were investigated, as shown in Figure 3. As shown in Figure 3a, the survey spectra of the f-CNTs, PABA, and PABA/f-CNTs presented C1s peaks at approximately 285 eV. The PABA and PABA/f-CNTs showed an N1s peak at about 400 eV. The presence of oxygen in the films may be attributed to the contamination during the film preparation and hydrolysis of PABA molecules [45,46]. The high-resolution spectrum of f-CNTs, as shown in Figure 3b, exhibited C1s peaks at 285.0, 286.0, 287.1, and 288.1 eV, corresponding to C–C(sp^3^) from the defect in the aromatic structure of CNTs, C–O, C=O, and O–C=O, respectively [13,47]. Figure 3c,d present the high-resolution spectra of C1s and N1s peaks of the electrochemically synthesized PABA and PABA/f-CNT films, respectively. The PABA and PABA/f-CNT films showed identical C1s peaks at 285.0, 286.0, 286.8, and 289.0 eV, which correspond to C=C, C–N, C-N+/C=N+, C=N, respectively, consistent with the PABA structure [15]. The peak ratio of C-N+/C=N+ to C-N increased significantly from 1.5 in the PABA to 2.2 in the PABA/f-CNTs, indicating an interaction between PABA and f-CNTs in the PABA/f-CNT composite. In the N1s spectra, identical peaks of –N=, –NH–, and –NH_2_– were observed at 400.9 and 402.3 eV [15,48,49,50]. The peak ratio of –N= to –NH– increased from 0.09 in the PABA to 0.13 in the PABA/f-CNTs, suggesting an interaction between the PABA and f-CNTs. The XPS results further confirm the successful fabrication of the PABA/f-CNT composite film in this study.

**Figure 4:**
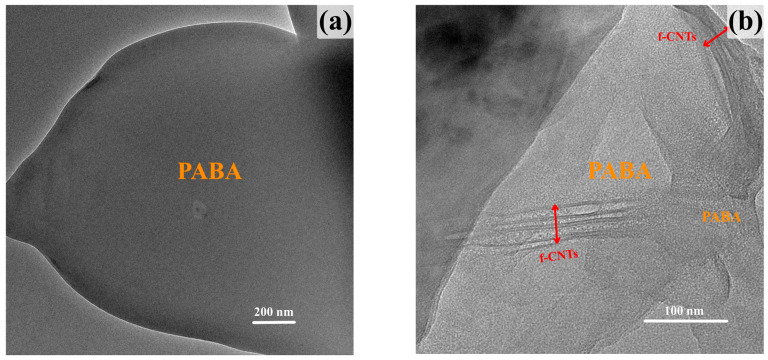
TEM images of (**a**) PABA and (**b**) PABA/f-CNT films.

The morphology of the obtained films was examined by TEM and SEM. The SEM images, as presented in Figure S1, show a regular particulate structure of the bare FTO, as shown in Figure S1a, which was subsequently covered with the uniformly electrochemically synthesized PABA and PABA/f-CNT composite films, as shown in Figure S1b,c. An SEM image of pristine f-CNTs is also shown in Figure S1d. No f-CNTs were observed after electropolymerization, as shown in the SEM image of the PABA/f-CNT composite film. In addition, Figure 4 shows TEM images of the flat surface of the electrochemically synthesized PABA (Figure 4a) and PABA/f-CNT composite (Figure 4b) films. The appearance of f-CNTs was clearly observed on the surface of the films. These observations again confirm the successful fabrication of the PABA and PABA/f-CNT composite films by electrochemical synthesis or electropolymerization.

### 3.2. Electrochemical Selective Dopamine Detection

Prior to use for selective electrochemical DA detection, the electroactivity of the electrochemically synthesized PABA and PABA/f-CNTs was investigated, as presented in Figure S2. The CV curves of the FTO, PABA, and PABA/f-CNT composite films in the PBS containing 0.5 mM K_3_Fe(CN)_6_ and 0.1 M KCl are shown in Figure S2a–c, with linear plots of the peak currents versus the square root of the scan rate shown in Figure S2d. This linear relationship implies that the electrode surface exhibited a linear diffusion-controlled process [12,13,14]. The broad CV curves with an average difference in anodic and cathodic peak potentials of about 0.31 V indicate the quasi-reversible process of the prepared films [12,51]. As shown in Figure S2e, the PABA/f-CNT composite exhibited a higher current response than the PABA. The electroactive surface area (A) of the FTO, PABA, and PABA/f-CNT electrodes was calculated from the Randles–Sevcik equation of these linear plots at the angular coefficient [12]. The FTO, PABA, and PABA/f-CNTs presented electroactive surface areas of 0.0222, 0.0249, and 0.0308 cm^2^, respectively. These results indicate that the addition of f-CNTs enhanced the electrochemical performance or electroactivity of the electrochemically synthesized PABA film [13,15]. This is probably due to the π–π interaction between the carboxylic group of the f-CNTs and the amino group of the PABA [15,39,52].

**Figure 5:**
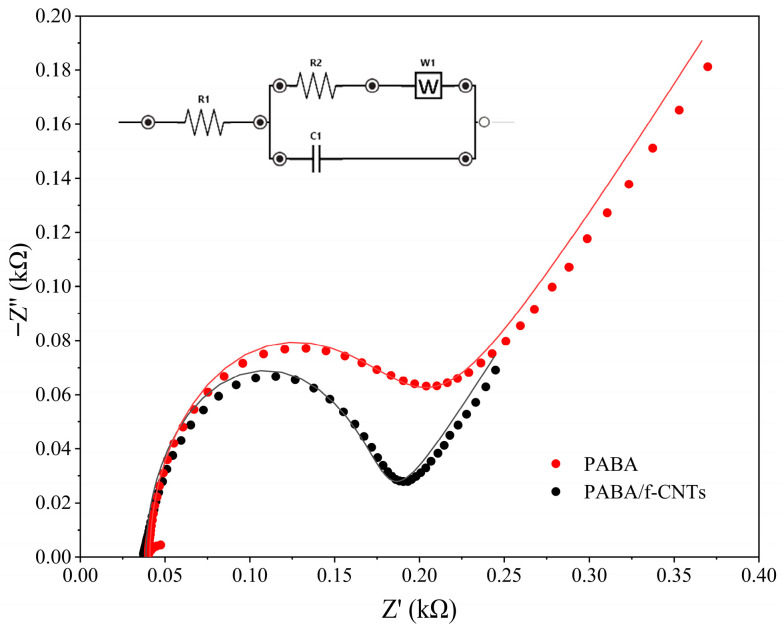
Nyquist plot with represented equivalent circuit model of the electrochemically synthesized PABA and PABA/f-CNT composite films in PBS containing 0.5 mM K_3_Fe(CN)_6_ and 0.1 M KCl at a frequency range of 1 Hz to 1 MHz at open circuit potential.

To investigate the effect of incorporating f-CNTs into the electrochemically synthesized PABA film, the electrochemical characteristics of the films were further studied by measuring impedance using EIS in PBS containing 0.5 mM K_3_Fe(CN)_6_ and 0.1 M KCl. Figure 5 represents the EIS spectra of the FTO, electrochemically synthesized PABA, and PABA/f-CNT electrodes at an open circuit potential and frequency range of 1 Hz to 1 MHz with the corresponding Randles equivalent circuit model. Semicircle portions of the obtained Nyquist plots were employed to calculate the charge or electron transfer resistance (Rct) of the electrochemically synthesized films, which were used as the working electrodes for selective electrochemical DA detection [53]. The smallest semicircle for the PABA/f-CNTs presented an Rct of 133.2 Ω, whereas the PABA presented an Rct of 141.7 Ω. The surface resistance of the PABA/f-CNTs was reduced due to the incorporation of f-CNTs in the film, which enhanced electron transfer and thereby increased the electroactivity of the electropolymerized film. Furthermore, the charge transfer rate (ks) of the films was calculated using the equation of ks = RT/n^2^F^2^RctC, where F = 96,485 C mol^−1^, R = 8.314 J mol^−1^ K^−1^, n is the number of electron transfer (n = 1), T = 298 K, and C is the concentration of K_3_Fe(CN)_6_ [54]. The ks values of the PABA and PABA/f-CNTs were found to be 3.756 × 10^−6^ and 3.996 × 10^−6^ cm/s, respectively. The ks of PABA/f-CNTs was greater than that of PABA, which could indicate increased conductivity activity. Hence, these results suggest that the incorporation of f-CNTs enhanced the electroactivity of the PABA, thereby improving its performance for the selective electrochemical detection of DA, which will be discussed further.

**Figure 6:**
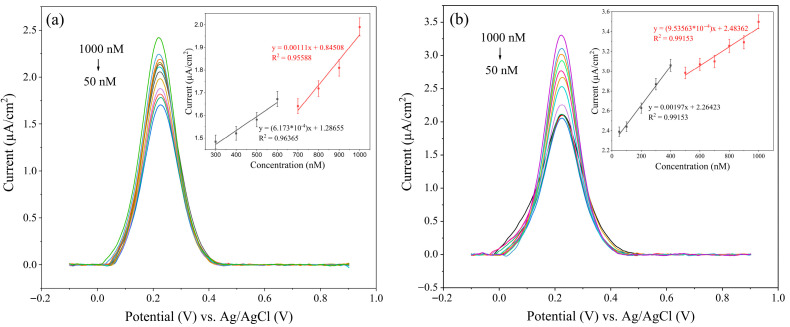
DPV responses (calibration curve, inset) of (**a**) PABA and (**b**) PABA/f-CNT composite films upon successive addition of DA (50–1000 nM) in the PBS containing 0.5 mM K_3_Fe(CN)_6_ and 0.1 M KCl.

The obtained electrochemically synthesized PABA/f-CNT composite film was used for label-free electrochemical detection of DA at concentrations of 50–1000 nM using the DPV technique in a potential range of −0.1 to 0.9 V at a scan rate of 5 mV/s in PBS containing 0.5 mM K_3_Fe(CN)_6_ and 0.1 M KCl. Figure 6 illustrates the DPV responses with linear plots of the response peak current versus concentrations, presented as calibration curves in the inset. The oxidation peaks in PBS containing 0.5 mM K_3_Fe(CN)_6_ and 0.1 M KCl appeared at about 0.25 V, which gradually enhanced with increasing DA concentrations. This observed peak was attributed to the oxidation of K_3_Fe(CN)_6_ in the PBS. With the DA addition, the DA oxidation peak appeared at the same potential with enhanced current density. This indicates that the PABA/f-CNTs and PABA catalyzed the oxidation of DA on the films [15]. This enhancement may result from the interaction between the quinoid structure of the PABA and the oxidized dopamine or dopaminechrome, which increased the observed peak currents upon DA addition [14,55]. The sensitivity for electrochemical detection of DA was derived from the slope of the obtained calibration curve [14,15]. The PABA film showed two linear ranges at 300–600 nM and 700–1000 nM (Figure 6a), with corresponding sensitivities of 0.617 and 1.11 µA·cm^−2^·µM^−1^, respectively. The limit of detection (LOD) and limit of quantitation ( LOQ) were 0.463 µM and 1.54 µM, respectively. In contrast, the PABA/f-CNT film (Figure 6b) presented sensitivities of 1.97 and 0.95 µA·cm^−2^·µM^−1^, with linear ranges of 50–400 nM and 500–1000 nM, respectively. The LOD and LOQ were 0.119 µM and 0.398 µM, respectively. These results demonstrate that the PABA/f-CNTs exhibited higher sensitivity in the lower concentration range with lower LOD and LOQ compared to the PABA. This electroactivity enhancement possibly arises from the hydrogen bonding between the carboxylic group (COOH) of the f-CNTs in the PABA/f-CNTs and the amino group of the DA molecule [15]. Table 1 presents a comparison of the results of this study, including the linear range, sensitivity, and LOD, with those of previous studies. It is possible that the differences in fabrication methods influenced sensor performance. For example, the SPANI/CNS film was fabricated by drop-casting a SPANI/CNS suspension onto a GCE electrode. This fabrication method could produce a thicker deposition layer that could enhance sensor performance. In contrast, in our work, the electropolymerization method resulted in the formation of a thinner film. Similarly, the PEDOT-LSG electrode exhibited a higher LOD value (0.33 μM) than that achieved in this work (0.119 μM), even though the PEDOT-LSG electrode possessed a three-dimensional macroporous network and a large electrochemically active surface area.

**Figure 7:**
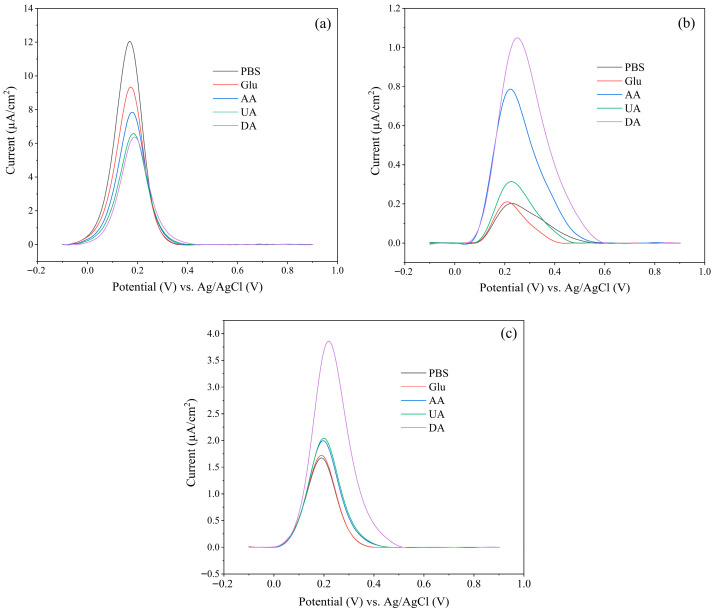
DPV curves for DA detection (1 mM) of the (**a**) FTO, (**b**) PABA, and (**c**) PABA/f-CNTs with the addition of the interferents (1 mM UA, AA, and Glu) in PBS containing 0.5 mM K_3_Fe(CN)_6_ and 0.1 M KCl.

The selectivity of the PABA/f-CNTs and PABA was evaluated with the addition of common interferents, including uric acid (UA), ascorbic acid (AA), and glucose (Glu). Other catecholamine-related compounds, such as serotonin, tyrosine, L-DOPA, epinephrine (EP), and norepinephrine (NE), which have a molecular structure similar to DA, may also interfere with DA detection. However, due to the bulky molecular sizes and low concentration of these molecules in urine, the effect of these molecules for DA detection in real samples may be negligible [13,14]. The DPV responses for DA detection with the addition of 1 mM interferents in PBS containing 0.5 mM K_3_Fe(CN)_6_ and 0.1 M KCl are presented in Figure 7. The DPV responses for DA detection with the addition of the interferents on the FTO substrate, as seen in Figure 7a, indicate that the current responses were comparable, with a decrease observed with the addition of interferents. For the PABA film, as shown in Figure 7b, the current responses were high for both AA and DA, with a moderate response for UA and a negligible response for Glu. However, the PABA/f-CNTs exhibited a markedly higher current response to DA compared with the interferents, as represented in Figure 7c. These selectivity results indicate that the PABA/f-CNTs exhibited good selectivity for label-free electrochemical DA detection.

The repeatability, reproducibility, and stability of the obtained PABA/f-CNTs and PABA were also studied. The reproducibility was performed upon detection of 0.1 mM DA in the PBS containing 0.5 mM K_3_Fe(CN)_6_ and 0.1 M KCl using three similar independent films. Figure S3a,b present the DPV responses for the reproducibility experiment of the PABA and PABA/f-CNTs, respectively. The PABA and PABA/f-CNT composite exhibited DPV peak responses consistent with the calculated relative standard deviation (RSD) values of 3.80% and 0.335%, respectively. These RSD values are acceptable, indicating that the electropolymerized films presented high reproducibility. The lower RSD of the PABA/f-CNT composite further confirms the electroactivity improvement of the film because of the incorporation of f-CNTs. In addition, the repeatability of the films was investigated via successive additions of 0.1 mM DA into the PBS containing 0.5 mM K_3_Fe(CN)_6_ and 0.1 M KCl for 10 cycles of DPV scans, as shown in Figure S4a for the PABA film and Figure S4b for the PABA/f-CNT composite film. The PABA and PABA/f-CNT films maintained stable responses for four and nine cycles, respectively. The improved repeatability of the PABA/f-CNT composite is likely due to enhanced mechanical stability conferred by the f-CNTs.

**Figure 8:**
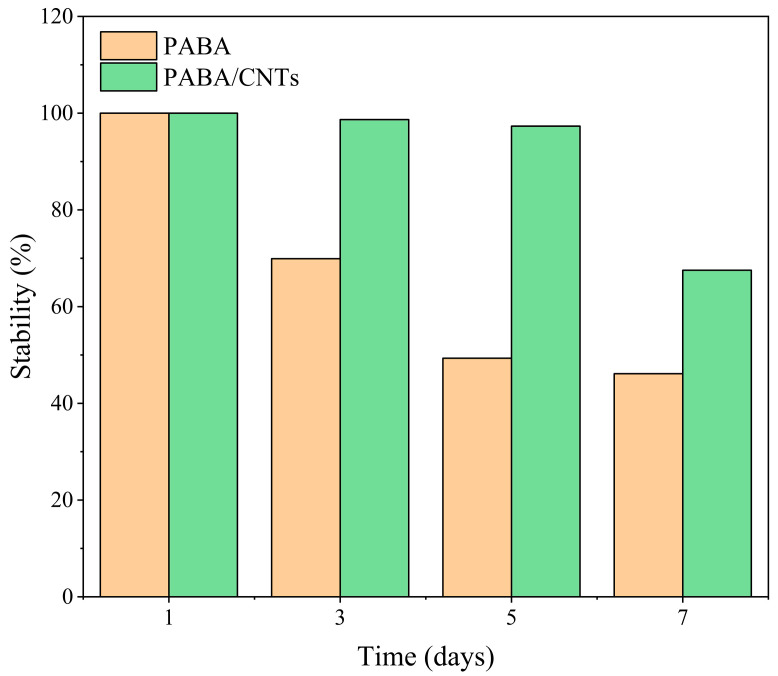
Stability histograms of the electropolymerized PABA/f-CNT and PABA films upon electrochemical DA detection.

In order to analyze the stability of the electropolymerized films, the DPV peak currents upon electrochemical detection of 0.1 mM DA were measured every two days for one cycle in PBS containing 0.5 mM K_3_Fe(CN)_6_ and 0.1 M KCl. The films were kept in the absence of humidity and stored at room temperature for up to 7 days. Stability histograms for evaluating the stability of the fabricated electropolymerized PABA/f-CNTs and PABA are presented in Figure 8. The DPV current response of the PABA film was dramatically decreased to below 80% after 3 days of storage and below 50% after 5 days of storage. In contrast, in the PABA/f-CNTs, the DPV current response of the composite film remained nearly unchanged after storage for 5 days. However, the DPV current response retained only 68% of the initial current response after 1 week. These results indicate that the incorporation of f-CNTs enhanced the stability and electroactivity of the PABA in the PABA/f-CNT film.

### 3.3. Artificial Urine Analysis

The electropolymerized PABA/f-CNTs and PABA were then applied as the working electrodes for electrochemical DA detection in artificial urine to verify the reliability of the obtained process in this study for application in real samples in the future. The electrochemical detection of DA (350, 550, and 750 nM for the PABA and 50, 150, 250, 350, 550, and 750 nM for the PABA/f-CNTs) was performed in 5% *v*/*v* of an artificial urine in PBS solution containing 0.5 mM K_3_Fe(CN)_6_ and 0.1 M KCl using the DPV technique at a scan rate of 5 mV/s under a potential range of −0.1–0.9 V for one cycle. The percentage recovery and RSD were calculated from the DPV peak current of each concentration using three similar independent electrodes. A high percentage of recovery with low RSD was obtained, as presented in Table 2, reflecting the excellent performance of the films regarding sensitivity, selectivity, reproducibility, repeatability, and stability. These results suggest that PABA/f-CNT and PABA electrodes are promising for future application in real samples.

## 4. Conclusions

A novel PABA/f-CNT composite thin film was successfully fabricated by electrochemical synthesis on a FTO glass substrate. The existence of the f-CNTs, which are located in the composite film, was investigated. The fabricated PABA/f-CNTs exhibited enhanced electrochemical DA detection performance in terms of sensitivity, selective, reproducibility, repeatability, and stability, compared to the PABA film. This enhancement was because the f-CNTs in the PABA/f-CNTs facilitated greater electron transfer and increased the electrochemical surface area. Although electrochemical synthesis produces thinner films than other fabrication techniques, such as drop-casting or electrospinning, the obtained electropolymerized PABA/f-CNTs resulted in a higher LOD value with higher sensitivity. Furthermore, the obtained PABA/f-CNTs exhibited strong performance for sensitive and selective label-free electrochemical DA detection in artificial urine with high reliability. In summary, the electrochemically synthesized PABA/f-CNT composite thin film exhibited potential for future application in real samples.

## Figures and Tables

**Table 1:** Comparable results of the presented study and previous studies for electrochemical DA detection.

Electrode	Linear Range (µM)	Sensitivity (µA·cm^−2^·µM^−1^)	LOD (µM)	References
PANI/CQDs	10–90	0.00802	0.1013	[12]
Electrospun PABA/f-CNTs	0.05–0.5	7.269	0.1554	[15]
I_2_-CMP-CNT-4	1–10	-	1.7	[56]
PEDOT-LSG	1–150	0.220	0.33	[57]
PANI-NF/Pt	62.5–603	-	33.30	[58]
SPANI/CNSs	0.50–1780	1.139	0.0152	[59]
PPy/CNTs	0.625–100	-	0.06	[60]
Electropolymerized PABA/f-CNTs	0.05–0.4 and 0.5–1	1.97 and 0.15	0.119	This work

Abbreviations: Conjugated microporous poly(arylene-ethynylene)-4 network (CMP-CNT-4); poly(3,4-ethylenedioxythiophene)-modified laser/graphene (PEDOT-LSG); polyaniline nanofiber-modified platinum electrode (PANI-NF/Pt); sulfonated polyaniline-decorated carbon nanosphere nanocomposites (SPANI/CNSs); poly(3-aminobenzylamine) (PABA).

**Table 2:** DA determination in artificial urine (n = 3).

Film	Added Concentration (nM)	Measured Concentration (nM)	Recovery (%)	RSD (%)
PABA	350	353	101	0.71
	550	571	103.9	1.10
	750	750	100	0.87
PABA/f-CNTs	50	47	94.4	2.08
	150	147	98.2	1.19
	250	247	98.9	1.75
	350	349	99.9	1.67
	550	551	100.3	0.24
	750	750	100	0.50

## Data Availability

The original contributions presented in the study are included in the article/Supplementary Materials, further inquiries can be directed to the corresponding author.

## Supplementary Materials

The following supporting information can be downloaded at https://www.mdpi.com/article/10.3390/polym17182539/s1: Figure S1: SEM images of (a) FTO, (b) PABA, and (c) PABA/f-CNT films and (d) f-CNTs. Figure S2: Cyclic voltammograms of (a) FTO, (b) PABA, and (c) PABA/f-CNT films with (d) linear responses of DPV peak currents and square root of scan rate. Figure S3: DPV responses for reproducibility study of (a) PABA and (b) PABA/f-CNTs. Figure S4: Differential pulse voltammograms for repeatability study of (a) PABA and (b) PABA/f-CNT films with addition of DA (0.1 mM) for up to 10 cycles.

## References

[1] Min J., Sempionatto J.R., Teymourian H., Wang J., Gao W. (2021). Wearable Electrochemical Biosensors in North America. Biosens. Bioelectron..

[2] Algov I., Feiertag A., Shikler R., Alfonta L. (2022). Sensitive Enzymatic Determination of Neurotransmitters in Artificial Sweat. Biosens. Bioelectron..

[3] Snider S.R., Kuchel O. (1983). Dopamine: An Important Neurohormone of the Sympathoadrenal System. Significance of Increased Peripheral Dopamine Release for the Human Stress Response and Hypertension. Endocr. Rev..

[4] Ohshiro K., Sasaki Y., Minami T. (2023). An Extended-Gate-Type Organic Transistor-Based Enzymatic Sensor for Dopamine Detection in Human Urine. Talanta Open.

[5] Li J., Zhao J., Wei X. (2009). A Sensitive and Selective Sensor for Dopamine Determination Based on a Molecularly Imprinted Electropolymer of *O*-Aminophenol. Sens. Actuators B.

[6] Suriyaprakash J., Huang Y., Hu Z., Wang H., Zhan Y., Zhou Y., Thangavelu I., Wu L. (2023). Laser Scribing Turns Plasticwaste into a Biosensor via the Restructuration of Nanocarbon Composites for Noninvasive Dopamine Detection. Biosensors.

[7] Dobryakova E., Genova H.M., DeLuca J., Wylie G.R. (2015). The Dopamine Imbalance Hypothesis of Fatigue in Multiple Sclerosis and Other Neurological Disorders. Front. Neurol..

[8] Albrecht D.S., MacKie P.J., Kareken D.A., Hutchins G.D., Chumin E.J., Christian B.T., Yoder K.K. (2016). Differential Dopamine Function in Fibromyalgia. Brain Imaging Behav..

[9] Feng Z.Y., Liu R., Jiang J.C., Meng L.Y. (2024). Villous 3D Nanoconfined Flexible Carbon Fibers-Based Electrode Toward Dopamine Electrochemical Detection. Diam. Relat. Mat..

[10] Silva L.I.B., Ferreira F.D.P., Freitas A.C., Rocha-Santos T.A.P., Duarte A.C. (2009). Optical Fiber Biosensor Coupled to Chromatographic Separation for Screening of Dopamine, Norepinephrine and Epinephrine in Human Urine and Plasma. Talanta.

[11] Maráková K., Piešťanský J., Zelinková Z., Mikuš P. (2020). Simultaneous Determination of Twelve Biogenic Amines in Human Urine as Potential Biomarkers of Inflammatory Bowel Diseases by Capillary Electrophoresis—Tandem Mass Spectrometry. J. Pharm. Biomed. Anal..

[12] Ratlam C., Phanichphant S., Sriwichai S. (2020). Development of Dopamine Biosensor Based on Polyaniline/Carbon Quantum Dots Composite. J. Polym. Res..

[13] Sriwichai S., Phanichphant S. (2022). Fabrication and Characterization of Electrospun Poly(3-Aminobenzylamine)/Functionalized Multi-Walled Carbon Nanotubes Composite Film for Electrochemical Glucose Biosensor. Express Polym. Lett..

[14] Panapimonlawat T., Phanichphant S., Sriwichai S. (2021). Electrochemical Dopamine Biosensor Based on Poly(3-Aminobenzylamine) Layer-By-Layer Self-Assembled Multilayer Thin Film. Polymers.

[15] Kaewda C., Sriwichai S. (2024). Label-free Electrochemical Dopamine Biosensor Based on Electrospun Nanofibers of Polyaniline/Carbon Nanotube Composites. Biosensors.

[16] Dube A., Malode S.J., Alodhayb A.N., Mondal K., Shetti N.P. (2025). Conducting Polymer-Based Electrochemical Sensors: Progress, Challenges, and Future Perspectives. Talanta Open.

[17] Ramanavicius S., Ramanavicius A. (2020). Conducting Polymers in the Design of Biosensors and Biofuel Cells. Polymers.

[18] Li J., Huang X., Shi W., Jiang M., Tian L., Su M., Wu J., Liu Q., Yu C., Gu H. (2021). Pt Nanoparticle Decorated Carbon Nanotubes Nanocomposite Based Sensing Platform for the Monitoring of Cell-Secreted Dopamine. Sens. Actuators B.

[19] Stern E., Wagner R., Sigworth F.J., Breaker R., Fahmy T.M., Reed M.A. (2007). Importance of the Debye Screening Length on Nanowire Field Effect Transistor Sensors. Nano Lett..

[20] Kaisti M. (2017). Detection Principles of Biological and Chemical FET Sensors. Biosens. Bioelectron..

[21] Prodromidis M.I. (2010). Impedimetric Immunosensors—A review. Electrochim. Acta.

[22] Tsai M.-Y., Creedon N., Brightbill E., Pavlidis S., Brown B., Gray D.W., Shields N., Sayers R., Mooney M.H., O’Riordan A. (2017). Direct Correlation Between Potentiometric and Impedance Biosensing of Antibody-Antigen Interactions Using An Integrated System. Appl. Phys. Lett..

[23] Zhang H.M., Li N.Q., Zhu Z.W. (2000). Electrocatalytic Response of Dopamine at a DL-Homocysteine Self-Assembled Gold Electrode. Microchem. J..

[24] Zhao H., Zhang Y.Z., Yuan Z.B. (2001). Study on the Electrochemical Behavior of Dopamine with Poly(sulfosalicylic acid) Modified Glassy Carbon Electrode. Anal. Chim. Acta.

[25] Awuzie C.I. (2017). Conducting Polymers. Mater. Today Proc..

[26] Namsheer K., Rout C.S. (2021). Conducting Polymers: A Comprehensive Review on Recent Advances in Synthesis, Properties and Applications. RSC Adv..

[27] Zhao F., Shi Y., Pan L., Yu G. (2017). Multifunctional Nanostructured Conductive Polymer Gels: Synthesis, Properties, and Applications. Acc. Chem. Res..

[28] Le T.H., Kim Y., Yoon H. (2017). Electrical and Electrochemical Properties of Conducting Polymers. Polymers.

[29] Nezakati T., Seifalian A., Tan A., Seifalian A.M. (2018). Conductive Polymers: Opportunities and Challenges in Biomedical Applications. Chem. Rev..

[30] Varol H.S., Herberger T., Kirsch M., Mikolei J., Veith L., Kannan-Sampathkumar V., Brand R.D., Synatschke C.V., Weil T., Andrieu-Brunsen A. (2023). Electropolymerization of Polydopamine at Electrode-Supported Insulating Mesoporous Films. Chem. Mat..

[31] Zhang M., Nautiyal A., Du H., Wei Z., Zhang X., Wang R. (2021). Electropolymerization of Polyaniline as High-Performance Binder Free Electrodes for Flexible Supercapacitor. Electrochim. Acta.

[32] Bhattacharyya A.S. (2024). Conducting Polymers in Biosensing: A Review. Chem. Phys. Impact.

[33] Ahmed Y.M., Eldin M.A., Galal A., Atta N.F. (2024). Electrochemical Sensor Based on PEDOT/CNTs-Graphene Oxide for Simultaneous Determination of Hazardous Hydroquinone, Catechol, and Nitrite in Real Water Samples. Sci. Rep..

[34] Cymann-Sachajdak A., Graczyk-Zajac M., Trykowski G., Wilamowska-Zawłocka M. (2021). Understanding the Capacitance of Thin Composite Films Based on Conducting Polymer and Carbon Nanostructures in Aqueous Electrolytes. Electrochim. Acta.

[35] Amiri M., Golmohammadi F., Safari M., Sadeq T.W. (2024). Electrochemical Synthesis of Polypyrrole Composite with Modified Gold Nanoparticles for Electrochemical Detection of Ascorbic Acid, Dopamine and Acetaminophen in Different Pharmaceutical Samples. Polym. Bull..

[36] Alahmadi N., El-Said W.A. (2023). Electrochemical Sensing of Dopamine Using Polypyrrole/Molybdenum Oxide Bilayer-Modified ITO Electrode. Biosensors.

[37] Shirodkar N., Cheng S., Seidel G.D. (2021). Enhancement of Mode I Fracture Toughness Properties of Epoxy Reinforced with Graphene Nanoplatelets and Carbon Nanotubes. Compos. Part B Eng..

[38] Quan D., Mischo C., Binsfeld L., Ivankovic A., Murphy N. (2020). Fracture Behaviour of Carbon Fibre/Epoxy Composites Interleaved By MWCNT- and Graphene Nanoplatelet-Doped Thermoplastic Veils. Compos. Struct..

[39] Demski S., Dydek K., Bartnicka K., Majchrowicz K., Kozera R., Boczkowska A. (2023). Introduction of SWCNTs as a Method of Improvement of Electrical and Mechanical Properties of CFRPs Based on Thermoplastic Acrylic Resin. Polymers.

[40] Wang X., Tang F., Cao Q., Qi X., Zhang H.P., Lin Z. (2020). Comparative Study of Three Carbon Additives: Carbon Nanotubes, Graphene, and Fullerene-C60, for Synthesizing Enhanced Polymer Nanocomposites. Nanomaterials.

[41] Iijima S. (1991). Helical Microtubules of Graphitic Carbon. Nature.

[42] Zhou Y., Zhou X., Ge C., Zhou W., Zhu Y., Xu B. (2019). Branched Carbon Nanotube/Carbon Nanofiber Composite for Supercapacitor Electrodes. Mater. Lett..

[43] Baughman R.H., Zakhidov A.A., de Heer W.A. (2002). Carbon Nanotubes—The Route Toward Applications. Science.

[44] Elaskalany M., Behdinan K. (2023). Effect of Carbon Nanotube Type and Length on the Electrical Conductivity of Carbon Nanotube Polymer Nanocomposites. Mater. Res. Express.

[45] Ahuja P., Ujjain S.K., Arora I., Samim M. (2018). Hierarchically Grown Nio-Decorated Polyaniline-Reduced Graphene Oxide Composite for Ultrafast Sunlight-Driven Photocatalysis. ACS Omega.

[46] Deng J., Wang T., Guo J., Liu P. (2017). Electrochemical Capacity Fading of Polyaniline Electrode in Supercapacitor: An XPS Analysis. Prog. Nat. Sci. Mater. Int..

[47] Wang H., Zhou A., Peng F., Yu H., Yang J. (2007). Mechanism Study on Adsorption of Acidified Multiwalled Carbon Nanotubes to Pb(II). J. Colloid Interface Sci..

[48] Marmisollé W.A., Gregurec D., Moya S., Azzaroni O. (2015). Polyanilines with Pendant Amino Groups as Electrochemically Active Copolymers at Neutral pH. ChemElectroChem.

[49] Fenoy G.E., Giussi J.M., Bilderling C., Maza E.M., Pietrasanta L.I., Knoll W., Marmisollé W.A., Azzaroni O. (2018). Reversible Modulation of the Redox Activity in Conducting Polymer Nanofilms Induced by Hydrophobic Collapse of a Surface-Grafted Polyelectrolyte. J. Colloid Interface Sci..

[50] Baba A., Mannen T., Ohdaira Y., Shinbo K., Kato K., Kaneko F., Fukuda N., Ushijima H. (2010). Detection of Adrenaline on Poly(3-Aminobenzylamine) Ultrathin Film by Electrochemical-Surface Plasmon Resonance Spectroscopy. Langmuir.

[51] Shrestha B.K., Ahmad R., Mousa H.M., Kim I.-G., Kim J.I., Neupane M.P., Park C.H., Kim C.S. (2016). High-Performance Glucose Biosensor Based on Chitosan-Glucose Oxidase Immobilized Polypyrrole/Nafion/Functionalized Multi-Walled Carbon Nanotubes Bio-Nanohybrid Film. J. Colloid Interface Sci..

[52] Gopal J., Muthu M., Sivanesan I. (2023). A Comprehensive Compilation of Graphene/Fullerene Polymer Nanocomposites for Electrochemical Energy Storage. Polymers.

[53] Lazanas A.C., Prodromidis M.I. (2023). Electrochemical Impedance Spectroscopy-A Tutorial. ACS Meas. Sci. Au.

[54] Megalamani M.B., Nandibewoor S.T. (2024). A Novel Electrochemical Strategy Using a Newly Developed Carbon-Based Sensor for the Detection of Antihypertensive Drug: Hydralazine Hydrochloride. Diam. Relat. Mat..

[55] Mondal R., Mukherjee N., Ahmed S.F. (2024). Ultrafast, Selective, and ppb Level In Vitro Electrochemical Sensing of Dopamine in a Simulated Interfering Environment: Comparative Study on the Effect of Carrier Type of Electrode Materials. ACS Appl. Electron. Mater..

[56] Paul J., Moniruzzaman M., Kim J. (2023). Framing of Poly(Arylene-Ethynylene) Around Carbon Nanotubes and Iodine Doping for the Electrochemical Detection of Dopamine. Biosensors.

[57] Xu G., Jarjes Z.A., Desprez V., Kilmartin P.A., Travas-Sejdic J. (2018). Sensitive, Selective, Disposable Electrochemical Dopamine 48Sensor Based on PEDOT-Modified Laser Scribed Graphene. Biosens. Bioelectron..

[58] Yu H., Pang Y., Tang J. (2015). Polyaniline Nanofiber Modified Platinum Electrode Used to Determination of Dopamine by Square Wave Voltammetry Technique. Int. J. Electrochem. Sci..

[59] Fu Y., Sheng Q., Zheng J. (2017). The Novel Sulfonated Polyaniline-Decorated Carbon Nanosphere Nanocomposites for Electrochemical Sensing of Dopamine. New J. Chem..

[60] Kan X., Zhou H., Li C., Zhu A., Xing Z., Zhao Z. (2012). Imprinted Electrochemical Sensor for Dopamine Recognition and Determination Based on a Carbon Nanotube/Polypyrrole Film. Electrochim. Acta.

