# Supplementary material for: Electrochemical Synthesis of Aminated Polyaniline/Multi-Walled Carbon Nanotube Composite for Selective Dopamine Detection in Artificial Urine
Source: Polymers (Basel). 2025 Sep 19;17(18):2539. doi: 10.3390/polym17182539 (PMC12473575; doi:10.3390/polym17182539)
Supplement: Supplementary file 1 [file polymers-17-02539-s001.zip › polymers-3816663-supplementary.pdf]

Electrochemical Synthesis of Aminated Polyaniline/Multi-Walled Carbon Nanotube Composite for Selective Dopamine Detection in Artificial Urine

Saengrawee Sriwichai [1,2,3,*] and Pimmada Thongnoppakhun [1]

[1] Department of Chemistry, Faculty of Science, Chiang Mai University, Chiang Mai 50200, Thailand;
thongnoppakhunpimmada@gmail.com
[2] Center of Excellence in Materials Science and Technology, Chiang Mai University, Chiang Mai 50200, Thailand
[3] Center of Excellence for Innovation in Chemistry (PERCH-CIC), Faculty of Science, Chiang Mai University, Chiang Mai 50200, Thailand
* Correspondence: saengrawee.s@cmu.ac.th

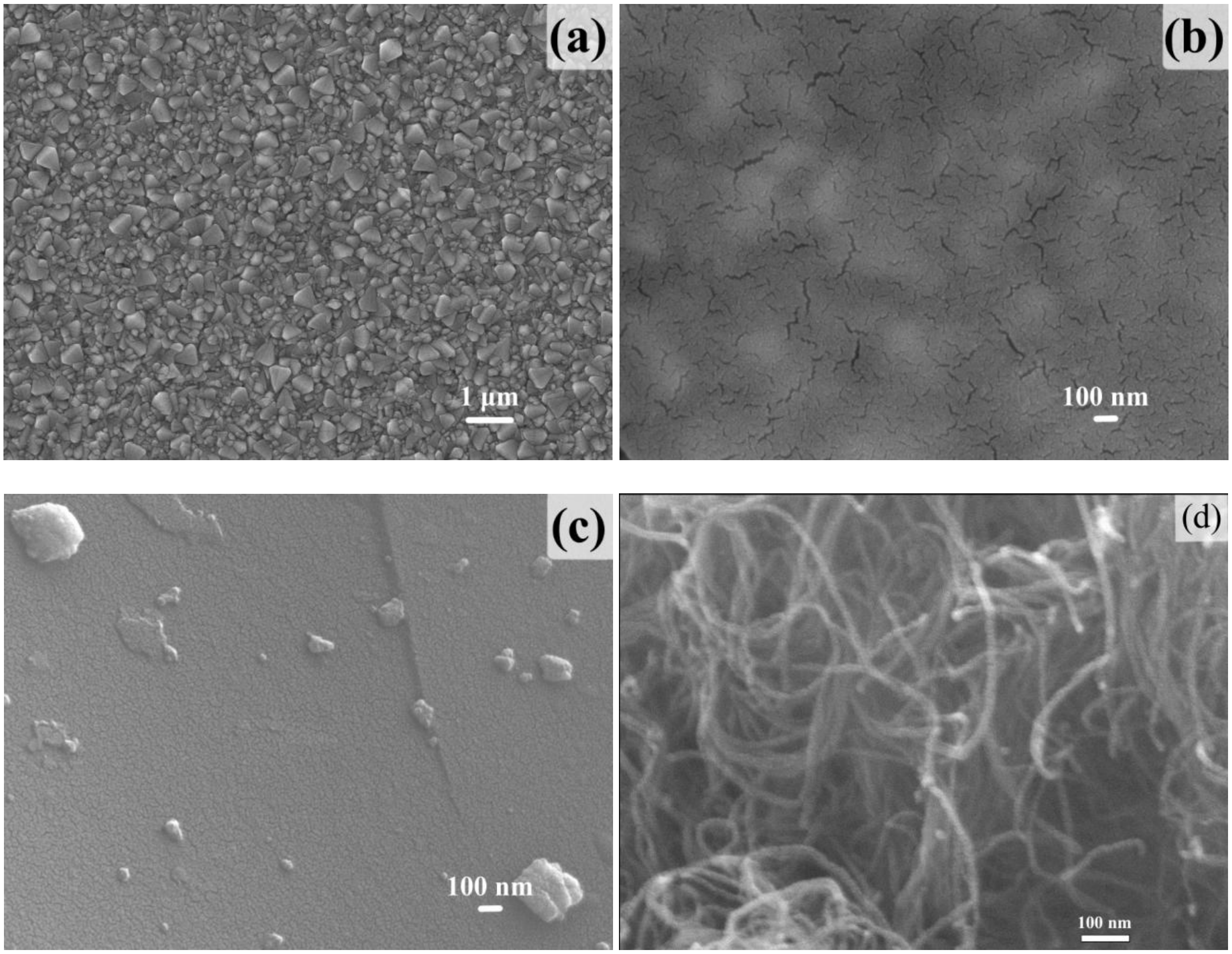

Figure S1. SEM images of (a) FTO, (b) PABA, (c) PABA/f-CNTs films and (d) f-CNTs.

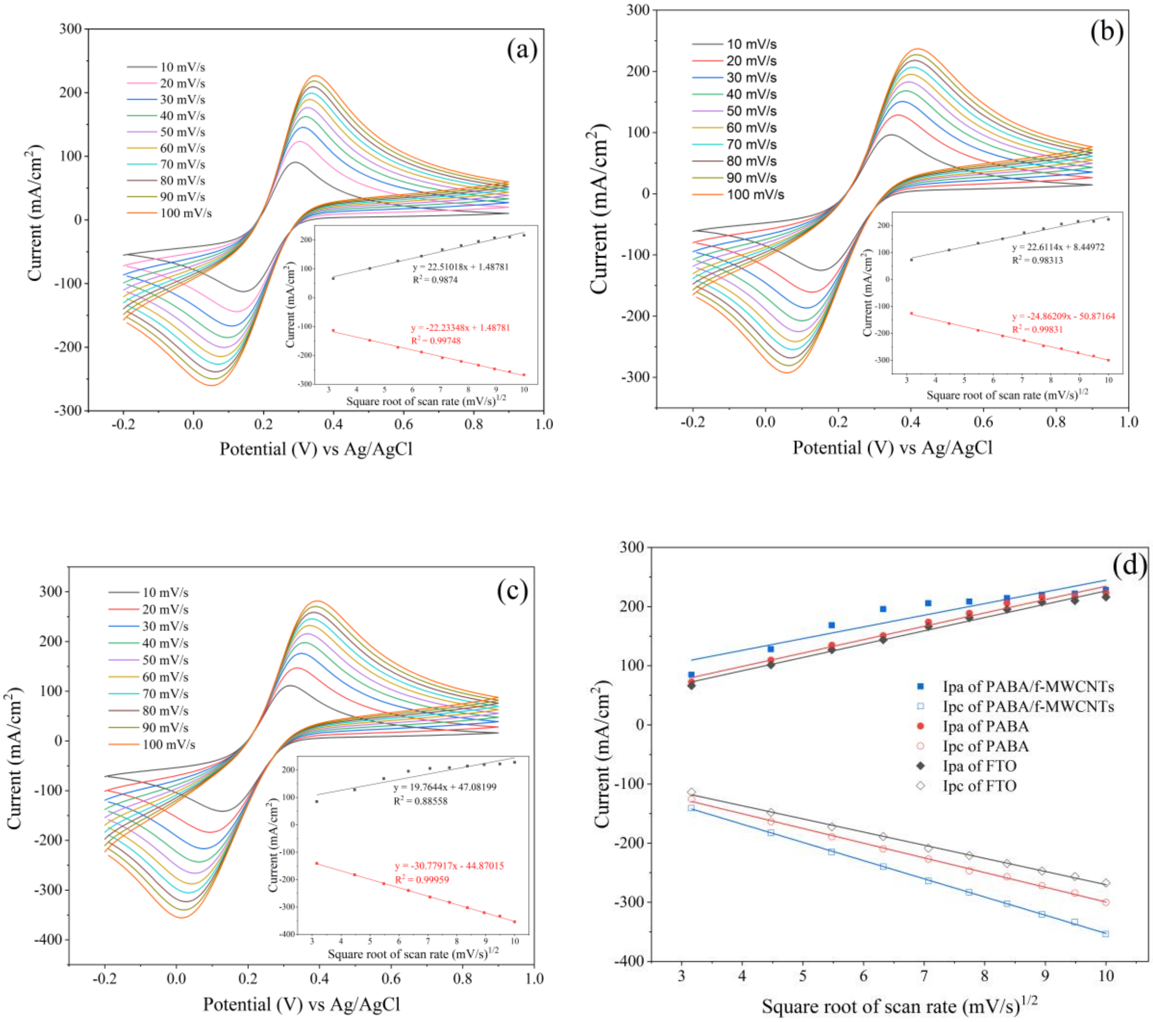

Figure S2. Cyclic voltammograms of (a) FTO, (b) PABA, and (c) PABA/f-CNTs films with (d) linear resposes of DPV peak currents and square root of scan rate.

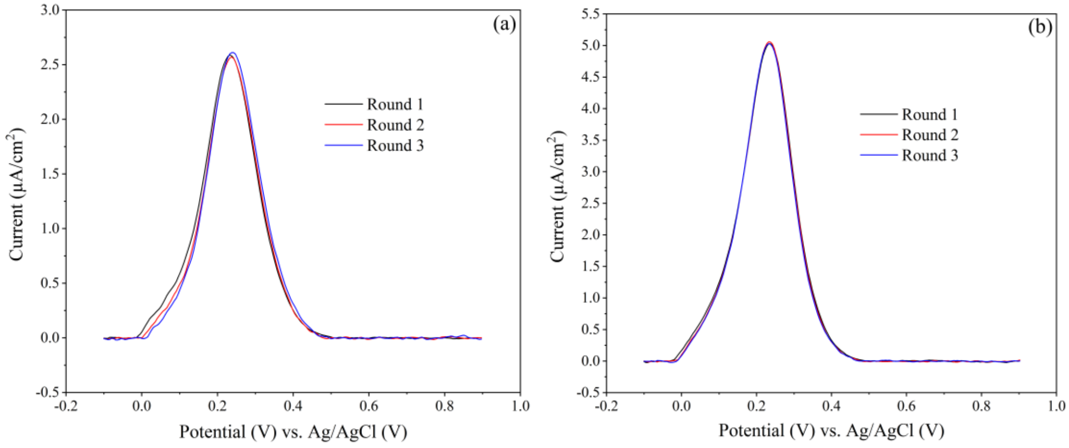

Figure S3. DPV responses for reproducibility study of (a) PABA (b) PABA/f-CNTs.

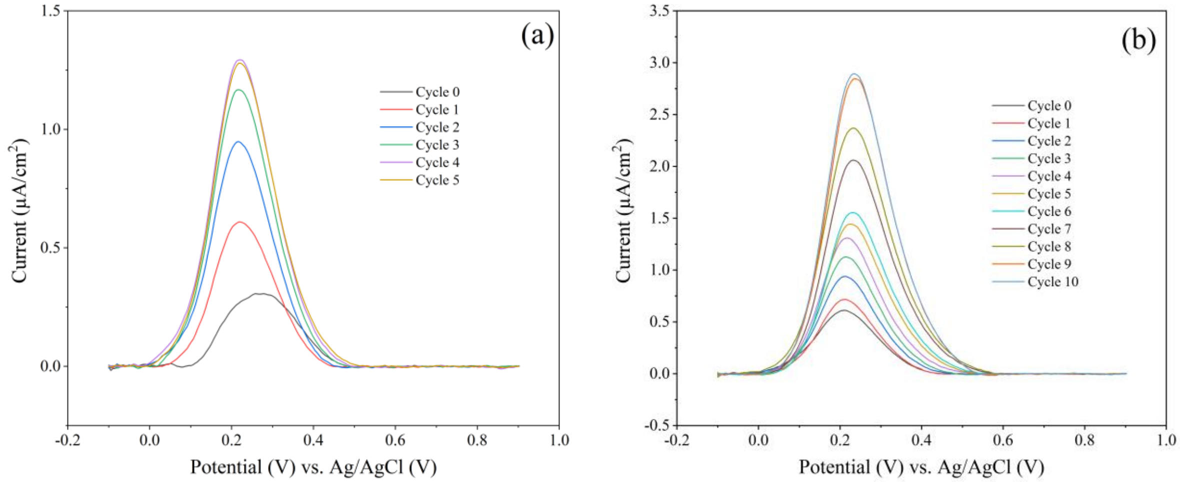

Figure S4. Differential pulse voltammograms for repeatability study of (a) PABA (b) PABA/f-CNTs films upon adding DA (0.1 mM) for up to 10 cycles.
